# Evaluation of CMR predictors of right ventricular remodelling in dilated cardiomyopathy

**DOI:** 10.1186/1532-429X-18-S1-P281

**Published:** 2016-01-27

**Authors:** Upasana Tayal, Simon Newsome, Ricardo Wage, Aamir Ali, Brian Halliday, Zohreh Farzad, Dudley J Pennell, Stuart Cook, Sanjay Prasad

**Affiliations:** 1 grid.439338.6Royal Brompton Hospital, London, United Kingdom; 2 grid.7445.20000000121138111Imperial College, London, United Kingdom; 3 grid.8991.9000000040425469XDepartment of Medical Statistics, London School of Hygiene and Tropical Medicine, London, United Kingdom; 4 grid.4280.e0000000121806431Duke National University Singapore, Singapore, Singapore

## Background

We have previously identified that right ventricular systolic dysfunction (RVSD), present in one third of patients with non-ischaemic dilated cardiomyopathy (DCM), is an independent predictor of all cause mortality and cardiac transplantation. CMR provides a robust and reliable way of quantifying RVSD.

The natural history of RVSD in DCM has not been formally evaluated. We sought to evaluate whether baseline left ventricular systolic function is predictive of the development of RVSD and whether progression of right-sided ventricular impairment is linked to progression of left-sided ventricular impairment.

## Methods

130 DCM patients (mean age 53.9 years, 65% male) underwent 2 cardiovascular magnetic resonance (CMR) studies, with a median interval of 2.7 years between studies (IQR 1.4-4.7 years). CMR was performed on a 1.5T Siemens scanner.

DCM was diagnosed on conventional criteria (increased LV end diastolic volume indexed to body surface area and reduced left ventricular ejection fraction (LVEF) compared with reference ranges normalised for age and gender; absence of significant underlying coronary artery disease). RVSD was defined as right ventricular ejection fraction (RVEF) <45%.

Data are presented as mean ± standard deviation. Correlation between interval change in RVEF and LVEF was assessed with Pearson's correlation test. ANCOVA was performed to identify independent predictors (Table 1) of the follow up RVEF adjusted for baseline RVEF. All statistical calculations were performed using R.

**Table 1:** Evaluated predictors of RV remodelling

CMR Imaging Predictors	Baseline LVEF, left ventricular mid wall fibrosis detected on LGE, mitral regurgitation, indexed left and right end diastolic and end systolic volumes.
Clinical Predictors	Age, gender, ethnicity, medication history (use of diuretic, beta blockers, ACE inhibitors, Aldosterone Antagonists), symptom status (NYHA class), resting heart rate, and comorbidities (hypertension and atrial fibrillation).

Evaluated predictors of RV remodelling (LVEF/RVEF=left/right ventricular ejection fraction, LGE= late gadolinium enhancement, NYHA= New York Heart Association symptom classification)

## Results

Mean baseline LVEF was 42% (± 12%) and RVEF 53% (± 16%). Thirty-two patients (25%) had RVSD at baseline, and 22 at follow up (17%). Mean follow up LVEF was 47% (± 13%) and RVEF 54% (± 14%).

When controlling for baseline RVEF, baseline LVEF was not predictive of follow up RVEF (p=0.19, 95% confidence interval -3% to 0.7%). When controlling for potential confounders, baseline LVEF remained non significant in predicting follow up RVEF.

**Figure 1:**
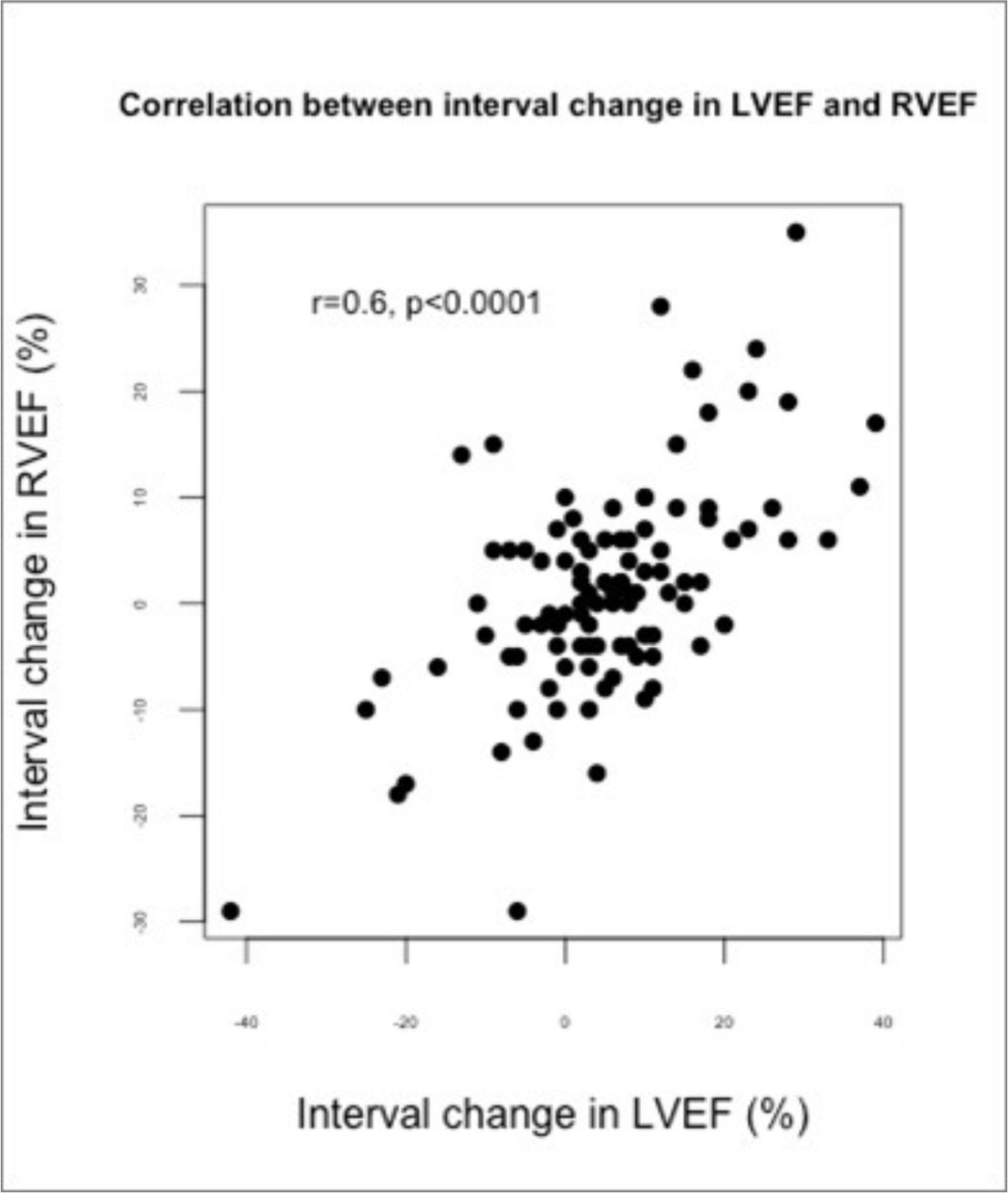
**Scatterplot showing the correlation between the interval change in LVEF and RVEF between CMR studies**. A positive interval change indicates reverse remodelling; a negative interval change indicates adverse remodelling.

The interval change in RVEF was strongly correlated with the interval change in LVEF between CMR studies (r = 0.6, p=<0.0001, figure 1). Controlling for baseline RVEF, the interval change in LVEF between studies was strongly predictive of follow up RVEF. For every 10% increase in LVEF between studies, the follow up RVEF would be 4.3% higher (p <0.0001, 95% confidence interval 3.1% to 5.4%). This remained highly significant even when adjusting for potential confounders(listed in Table 1).

## Conclusions

These data show no evidence that progression of RVSD in DCM is dependent on baseline left ventricular systolic function.

However, adverse or reverse remodelling of RV function mirrors the change in LV function.

This suggests therefore that patients with significant LV impairment but normal RV function at baseline may not necessarily develop RVSD. However if LV function improves or deteriorates then RV function is likely to follow a similar course.

